# Interactive Effects of *Epichloë* Endophyte, Dormancy-Breaking Treatments and Geographic Origin on Seed Germination of *Achnatherum inebrians*

**DOI:** 10.3390/microorganisms9112183

**Published:** 2021-10-20

**Authors:** Yaqi Chen, Kaiqi Su, Chunjie Li, James F. White

**Affiliations:** 1 State Key Laboratory of Grassland Agro-Ecosystems, Key Laboratory of Grassland Livestock Industry Innovation, Ministry of Agriculture and Rural Affairs, Engineering Research Center of Grassland Industry, Ministry of Education, Gansu Tech Innovation Center of Western China Grassland Industry, Center for Grassland Microbiome, College of Pastoral Agriculture Science and Technology, Lanzhou University, Lanzhou 730000, China; chenyq19@lzu.edu.cn; 2 State Key Laboratory of Grassland Agro-Ecosystems, National Demonstration Center for Experimental Grassland Science Education, College of Pastoral Agriculture Science and Technology, Lanzhou University, Lanzhou 730000, China; sukq19@lzu.edu.cn; 3 Department of Plant Biology, Rutgers University, New Brunswick, NJ 08901, USA; jwhite3728@gmail.com

**Keywords:** internal hormone, soluble sugar, gibberellin, ABA, local adaptation

## Abstract

Background: the cool-season grass *Achnatherum inebrians* (drunken horse grass) is an important species in the northwest grasslands of China. This grass engages in a symbiotic relationship with *Epichloë* endophytes, which affect host plants by increasing growth, repelling herbivores, and increasing tolerance to stressful environments. Methods: in this work, we evaluated the interaction effects of the endophyte on various dormancy-breaking treatments on A. *inebrians* seeds from six different locations. We used both endophyte-infected plants and noninfected plants and applied four dormancy-breaking methods to test germination. Results: our results showed that the germination rate of endophytic *Achnatherum inebrians* seeds from the Xiahe site (with highest altitude) was significantly higher than that from other sites when water soaking was applied (*p* < 0.05). Endophytic seeds had a greater germination rate, and soluble sugar, indole acetic acid (IAA), and gibberellin (GA) contents, under any condition. There was a significant interaction among the method, endophyte status, and origin regarding germination (*p* < 0.001); particularly, the effects of warm water soaking and endophyte infection on the germination of seeds from the Xiahe site was significant (*p* < 0.05). Conclusions: the infection of *Epichloë* endophyte is able to increase the content of soluble sugar, IAA, and GA, and stimulate the seed germination of A. *inebrians*.

## 1. Introduction

Seed dormancy prevents seeds from germinating, which protects plants from development in an unfavorable season [1]. Numerous studies proved that seed dormancy is caused by either seed coat hardness (physical) or embryo dormancy (physiological) [2,3]. Physical dormancy from seed drying causes the seed to remain dormant until sufficient water is available [4]. Physiological dormancy could be broken and achieved by removing the seed coat or surrounding tissues [5]. Another method to break dormancy is to add hormones, such as gibberellin (GA) and abscisic acid (ABA), which also promotes germination [2,6,7,8]. The seed is able to adjust the composition and deposition of both GA and ABA internally so that the seed remains dormant or germinates [9]. Studies suggest that ABA has a positive regulating effect on seed dormancy. Kucera [10] found that increased ABA content prevents germination, and internally produced ABA induces dormancy. A balance between GA and ABA is a critical factor controlling dormancy or germination [11]. In addition, Fennimore and Foley [12] suggested the prerequisites of dormancy termination are the breakdown of hormone balance, and GA plays a key role in seed germination. Indole acetic acid (IAA) as a growth regulator controls growth in the seed embryo [11]. Seed soluble sugar and starch substances provide essential nutrition for embryo development [13]. Soluble sugars in seeds are critical for embryo development [14]. Sugar is the main energy source for germination and further growth [15]. High concentrations of carbohydrate reduce chilling injury [16]. Germination is also influenced by the climate of geographic origin, in particular, mean annual temperature. Low elevation, warm climate, and rainfall promote seed germination. [16] found that *Achnatherum inebrians* from high altitudes with lower temperatures had higher germination rates than seed from warmer, low-elevation arid grasslands. Endophyte presence in seeds also affects the growth of plants and seed germination [17].

Drunken horse grass (*Achnatherum inebrians*) is one of the perennial toxic grasses in the northwest grassland of China [18,19]. Due to the presence of alkaloids produced by an endophytic fungus, livestock may be poisoned [20]. The study of drunken horse grass symbiosis began when Bruehl [21] isolated the fungus. Nan and Li [22] found two species and named them *Epichloë gansuensis* and *Epichloë inebrians*. The fungal endophyte is an example of an association between the plant and fungus that results in a positive outcome [23]. One feature of this endophyte is its intercellular growth inside plant tissues where it has a stable environment and essential nutrients [24]. Previous studies showed that infection by *Epichloë* species promoted adaptation to drought, cold, heavy metal, and other environmental stresses [18,19,25,26]. In the United States and New Zealand, numerous studies focused on the relationship between endophytes and tall fescue (*Festuca arundinacea*) and perennial ryegrass (*Lolium perenne*), which results in the tolerance and resistance of hosts to biotic and abiotic stresses [24,27,28,29,30]. The Northwest Plateau of China is undergoing severe soil erosion, with barren land and reduced forest cover, which leads to a decline in ecological functions. Water use efficiency is an important determinant of sustainable agricultural development in grasslands. The grass–endophyte relationship plays a role in improving water-use efficiency under drought conditions [31]. Yao [32] showed that drunken horse grass endophytes cause plants to competitively exclude other competitor plant species. The distribution and frequency of endophyte-infected (E+) drunken horse grass is more extensive than non-infected (E−) grass individuals in semi-arid and arid grasslands in Northwest China [18]. The interaction between fungal endophyte genus *Epichloë* and its host plants represents a unique model for examining ecological and biological benefits in fungal endophyte–plant interactions [23].

Studies showed that *Epichloë* endophytes have positive effects on host growth. However, far too little attention was paid to the mechanism by which fungal endophytes break seed dormancy in drunken horse grass. The central thesis of this paper is to investigate the interactive effect of *Epichloë* endophyte and various dormancy-breaking methods in A. *inebrians* from different origins. There are three primary aims of this study: 1. to investigate the *Epichloë* endophyte promotion of seed germination; 2. to evaluate the effects of dormancy-breaking methods on A. *inebrians* seed germination; 3. to compare levels of germination in seeds from various geographic origins with different climates.

## 2. Materials and Methods

### 2.1. Seed Materials

Seeds of A. *inebrians* were collected from six climatically different natural sites in July 2019 (Table 1). Half of the collected seeds were soaked into 200-times-diluted thiophanate methyl for 6 h to eliminate the endophyte [33] and labeled as E + 1 (primary generated endophyte-infected seeds) and E − 1 (primary generated endophyte-noninfected seeds). At each site, 15 E + 1 and E − 1 seeds were sown in seedling pots (65 × 28 × 65 mm), and this was repeated 3 times per site at Lanzhou University. Pots were arbitrarily placed in a greenhouse with a diurnal cycle of 25 °C and 20 °C day and night air temperatures, respectively. At the end of the growing stage, second-generation seeds were collected from each E + 1 and E − 1 plant and tested microscopically by aniline blue-lactic acid [34]. Then, they were labeled as E+ and E− seeds and stored at 4 °C until the next experiment. 

### 2.2. Breaking Dormancy and Germination Experiment

Mechanical, thermal, and chemical treatments were compared in terms of their relative effectiveness in breaking the seed dormancy of drunken horse grass seeds. Mechanical treatment was achieved by a needle pin, which was used to tear the seed coat without piercing the embryo; thermal treatment was completed by soaking in warm water (25 °C) for 30 min; chemical dormancy break was accomplished by soaking in 98% H_2_SO_4_ for 30 min [35] and in exogenous GA 1 part per million (ppm) for 20 min [36]. Intact E+ and E− seeds’ different sites without any treatments were used as control seeds (CK) (Table 2). To determine the optimum breaking method, 50 replications of 6 origins of E+ and E− seeds of 4 different treatments were placed in Petri dishes with two layers of filter papers. Each Petri dish contained 100 seeds. Petri dishes were arbitrarily laid flat into growth chambers (LRG-250-LED, China)—25 °C, diurnal cycle of 12 h day and night, and 35% humidity. The number of germinated seeds was recorded daily until no further germination was observed. Germination was considered as achieved when the radicle protrusion was approximately 2 mm. Experiments were continued for 30 days. The final germination rate was calculated on the basis of the total number of filled seeds.

### 2.3. Determination of Germination Rate

Germination was monitored daily, and germinated seeds were counted and removed from Petri dishes. Germination was considered as achieved when the radicle protrusion was approximately 2 mm. Experiments were continued for 30 days. The final germination percentage (FGP) was calculated on the basis of the total number of filled seeds. Germination rate = total germinated seed/total seed number × 100. The relative germination rate was calculated by subtracting the germination rate of controls.

### 2.4. Determination of Soluble Sugar

The soluble sugar content was determined by the sulfuric acid-anthrone color method [37]. Treated and untreated seeds were ground in liquid nitrogen. Aliquots of 0.1 g of each sample were homogenized in 5 mL 95% (*v*/*v*) ethanol. After centrifugation, 1 mL supernatant was mixed with 4 mL anthrone reagent and heated in a boiling water bath for 10 min. Absorbance was recorded at 620 nm after cooling. The amount of sugar was determined by the standard curve prepared from glucose.

### 2.5. Determination of Internal Hormone Levels

High-performance liquid chromatography (HPLC) was applied to test internal hormones, GA, IAA, and ABA [38]. American Waters Acquity Arc type high-performance liquid chromatography (Milford, MA, USA) was used for determination. The chromatographic conditions were chromatographic column: Symmetry C18 column (4.6 mm × 250 mm, 5 μm); mobile phase: 10% methanol + 90% φ = 0.1% phosphoric acid; flow rate: 1.0 mL/min; injection volume: 10 μL; detection wavelength: 254 nm; column temperature: 30 °C. We ground the seeds with liquid nitrogen in a dark environment, accurately weighed 2.0 g, washed them with 5 mL 80% chromatographic methanol 3 times in a test tube, wrapped them in aluminum foil paper and placed them into a refrigerator at 4 °C for 12 h extraction. We shook the mixture once every hour to dissolve the hormone in the organic phase. After shaking, we centrifuged the mixture at 4 °C 8000 r/min for 10 min; drew the supernatant into a 10 mL centrifuge tube; and extracted the filter residue twice, with 80% chromatographic methanol (shaking and mixing each time) and the addition of 2.5 mL each time, before soaking for 1 h; and finally, we combined the filtrate and diluted to 10 mL. We took 2 mL in a vacuum centrifugal concentrator and rotary evaporation (38 °C) until dry. We reconstituted it with 1.0 mL of 50% chromatographic methanol and passed it through a 0.22 μm organic phase microporous filter membrane to a sample bottle for testing.

### 2.6. Data Analysis

A three-way fixed effects ANOVA (*p* < 0.05) was performed to evaluate the effects of endophyte infection status (E+ and E−), treatments (M1, M2, M3, and M4), original habitat and their interactions on cumulative seed germination. The means were compared using Tukey’s test at the 95% confidence level. The significant difference in endophyte status (E+ or E−) in seed germination under different treatments was tested with a posthoc test with multiple comparisons of the mean analysis following one-way ANOVA. The difference between E+ and E− seed germination under the same treatment was tested by an independent t-test (*p* < 0.05). Prior to the analysis, the germination percentage data were arcsine transformed to meet the assumption of homogeneity of variance in the ANOVA. All of the analyses were performed using the IBM Statistical Product and Service Solution (SPSS) software (version 19.0; SPSS China, Shanghai, China).

## 3. Results

### 3.1. Germination Rate

Germination rates of the untreated controls were relatively high and ranged from 78–87 % for E− seeds and 83–87 % for E+ seeds depending on the origin of seed (see Figure S1). 

### 3.2. Physiological Elements

**Figure 1:**
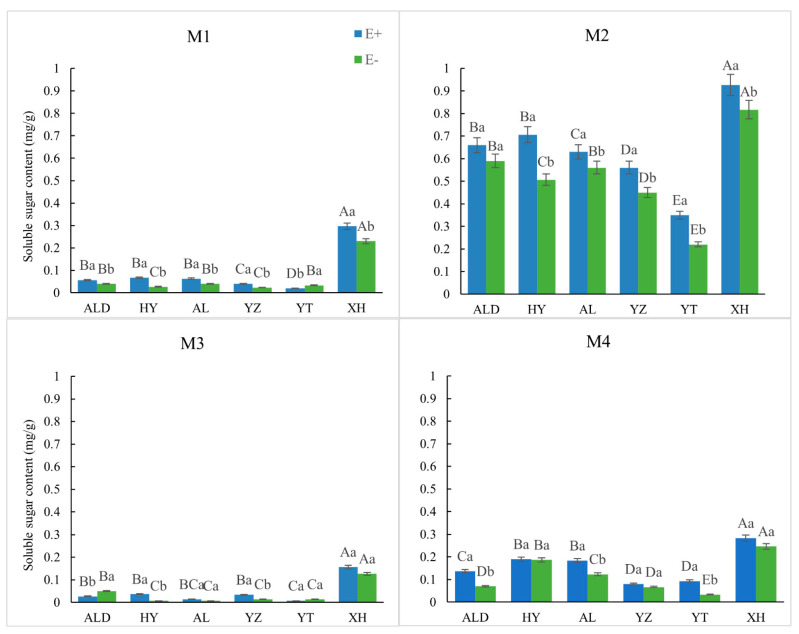
Relative soluble sugar content of infected (E+) and noninfected (E−) *Achnatherum inebrians* from different treatments on an altitude (origin) gradient. Different capital letters indicate mean significant difference (*p* < 0.05) in endophyte status (E+ or E−) in seed germination under different origin. Different lowercase letters indicate mean significant difference (*p* < 0.05) in pair-wise comparisons of endophyte status (E+ and E−) in seed germination in same origin.

Overall, the relative content of soluble sugar under the M2 treatment was different from that under the other treatments (*p* < 0.001). Pair-wise comparisons of seed-germination were made between seeds with different endophyte statuses (E+ and E−) of the same origin. (*p* < 0.05) (Figure 1). 

**Figure 2:**
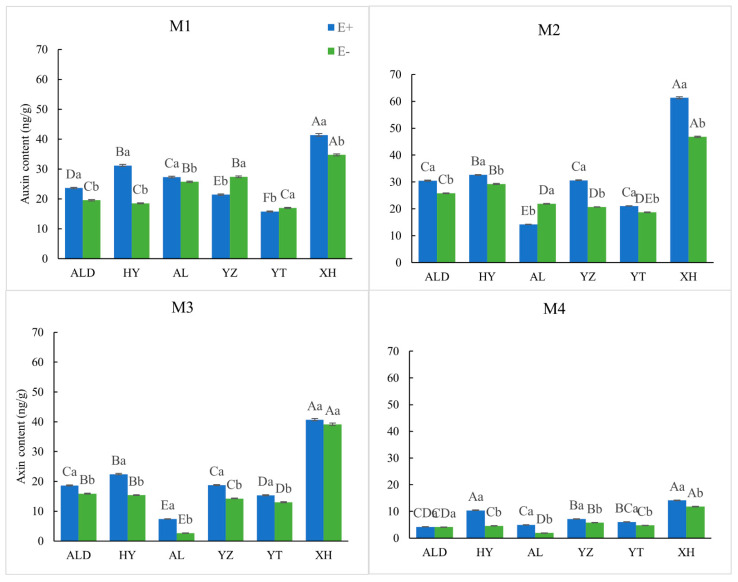
Relative auxin content of infected (E+) and non-infected (E−) *Achnatherum inebrians* from different methods on an altitude (origin) gradient. The different capital letters indicate the mean significant difference (*p* < 0.05) in endophyte status (E+ or E−) in seed germination under different origin. Different lowercase letters indicate the mean significant difference (*p* < 0.05) in the pair-wise comparisons of endophyte status (E+ and E−) in seed germination in the same origin.

Except for E− seeds from YZ and YT under M1 treatment, all E+ seeds appeared to have a higher relative auxin content than E− seeds (Figure 2). Both E+ and E− seeds under M2 treatment showed a significant (*p* < 0.05) great relative soluble sugar content than other treatments, and all results were lager than 0. Each break-dormancy treatment was able to increase the germination. 

**Figure 3:**
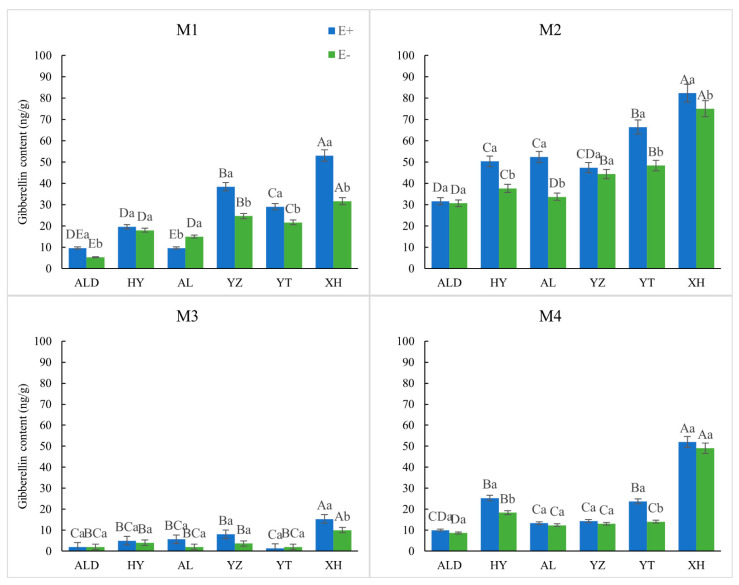
Relative gibberellin content of infected (E+) and non-infected (E−) *Achnatherum inebrians* from different treatments on an altitude (origin) gradient. The different capital letters indicate the mean significant difference (*p* < 0.05) in endophyte status (E+ or E−) in seed germination under different origin. The different lowercase letters indicate the mean significant difference (*p* < 0.05) in the pair-wise comparisons of endophyte status (E+ and E−) in seed germination in the same origin.

Except for E− seeds from AL, which under M1 treatment, all E+ seeds showed a higher relative GA content than E− seeds (Figure 3). Both seed types under M2 treatment showed a significantly greater relative GA content than under other treatments, and all treatment results were positive compared to the CK condition.

**Figure 4:**
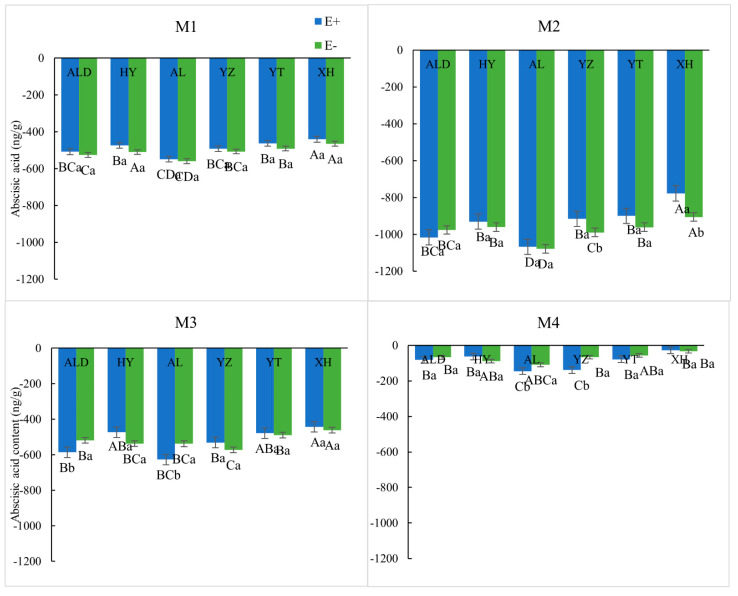
Abscisic acid content of infected (E+) and noninfected (E−) *Achnatherum inebrians* from different methods on an altitude (origin) gradient. Different capital letters indicate mean significant difference (*p* < 0.05) in endophyte status (E+ or E−) in seed germination under different origin. Different lowercase letters indicate mean significant difference (*p* < 0.05) in pair-wise comparisons of endophyte status (E+ and E−) in seed germination in same origin.

The M2 treatment gave the greatest reduction in ABA content relative to the controls; all E+ seeds had a significantly lower ABA content increase than E− seeds (*p* < 0.05). ABA was higher under CK conditions than for the treated seeds, although differences were relatively small for M4 compared with M1–M3. All treated seeds showed a negative result when compared to that of untreated seeds (Figure 4).

### 3.3. Interactive Effects

On average, the interaction effect of origin, different breaking dormancy treatment and endophyte was significant for all indicators (*p* < 0.05) (Table 3). Two-way ANOVA analysis suggested that the origin*method, origin*endophyte, and method*endophyte were significant (*p* < 0.001). The germination rate and gibberellin had significant effects under interactive effect of origin, method, and endophyte (*p* < 0.05). On the other hand, there was no effect of various methods on either the soluble sugar or abscisic acid content inside plants (Table 3).

## 4. Discussion

The current study showed that *Epichloë* endophyte-infected *Achantherum inebrians* seeds collected in the Xiahe site under warm water soaking had the highest germination rate. The soluble sugar, IAA, and GA contents were increased, and stimulated the seed germination of *A*. *inebrians*.

### 4.1. Effects of Endophyte on Achnatherum inebrians Seed Germination

Early reported studies emphasized the importance of seed dormancy as a strategy of plants to adapt to adversity and protect species regeneration [39]. Seed dormancy has an important ecological significance and can effectively regulate the spatial and temporal distribution of seed germination [40]. We found the infected endophyte is able to significantly (*p* < 0.05) increase *Achnatherum inebrins* seed germination rate under any breaking dormancy method and any geographic origin (*p* < 0.05). Our study findings are in accordance with previous reported studies. Li [22] and Nan et al. [9] claimed that *Epichloë* endophyte-infected grass had increased growth, higher resistance to disease and showed stronger vigor under environmental stress compared to that of noninfected plants. Furthermore, Chen et al. [41] and Zhang et al. [19] found infected drunken horse grass had a higher resistance to multiple oxidative stresses. Miransari and Smith [11] highlighted that IAA was important evidence in establishing polarity, while GA played a crucial role in promoting embryo growth. Early germination was associated with the increase in IAA and GA amounts, and the inhibition of ABA production. Our results were also similar to those of Miransari and Smith [11], who showed that endophytic-infected seeds had a higher content of IAA, GA, and soluble sugar, and a lower content of ABA, which might be due to the possible hypothesis that endophytes promote internal hormones to stimulate the germination rate. We found that E+ seeds from any origin had a greater germination rate under the ‘control treatment’, which might lead one to speculate that endophytes would stimulate germination. However, there was a significant interaction between the origin and treatments of seeds (*p* < 0.05), suggesting that treatment effects were different for different locations, which corroborates similar findings by Bao et al. [17]. The current study proved that *Epichloë* endophyte significantly enhanced the seed germination of drunken horse grass, under either a low or high temperature, which justified the statement that endophyte increases competitive advantages [42,43].

### 4.2. Effects of Geographic Origin on Seed Germination

The Xiahe site has the highest elevation and lowest temperature range and showed a significantly higher seed germination rate than other origins. Koornneef et al. [44] reported that temperature and altitude are important factors for species distribution and crop reproduction under natural environmental change. Our experimental site (Xiahe) had a relatively cold atmosphere, which promotes seed germination genes. Bao et al. [17] found that high-altitude plants had a greater germination rate. Previous studies confirmed that plants from low temperature alpine regions have a higher germination rate than plants from arid regions. Endophyte-infected seeds can survive under a wide range of temperature conditions, and have the ability to endure harsh or suboptimum environments. It is hypothesized that mother plants have strong effects on seed/plant progeny, and *Achnatherum inebrians* is able to survive under harsh environments due to traits that are passed down from mother plants [45,46]. In addition to dormancy-breaking methods and endophyte status, we observed the effects of genetic basis and background elements from different origins on second-generation seed germination. This is due to the fact that mother plants adapt to local environment. We tested the germination rate of all materials at room temperature with stable humidity.

### 4.3. Effects of Dormancy Breaking Treatments on Seed Germination

Our result showed that under the M2 condition, the germination rates of both E+ and E− seeds were higher than other treatments; all germination rates were better than those of the CK condition for all geographic origins. We speculated that the dormancy of the *Achantherum inebrains* seed was due to the lack of imbibition, or due to chilling. When under needle treatment or 98% H_2_SO_4_ treatment, there was a scarcely greater germination rate compared to the CK condition, and GA solution soaking also had little effect; hence, we strongly suggested M2. Soitamo et al. [46] found that the colder the climate, the higher the ABA content; this conclusion would explain the increased amount of ABA in the experiment involving seeds from the Alxa League desert, where temperatures usually remain cool. ABA plays an active role in inducing seed dormancy and maintaining the dormancy state. A low ABA content during seed development causes primary dormancy in mature seeds, while too much ABA will enhance the secondary dormancy of seeds and delay germination [11]. In our study, we did not have direct data to prove the effect of GA and ABA on germination; however, according to the comparison of the trends, we suspect that there was a positive correlation between GA and germination, and there was a negative correlation between ABA and the germination rate. Bethke et al. [47] and Kucera et al. [10] proved that the endogenous hormones and external environment of seeds co-regulate seed dormancy and germination. ABA resynthesizes when the dormant seed blots, thus keeping the seed in a dormant state. The higher IAA, GA, and soluble sugar, but lower ABA and endophyte infection, promote seed germination, possibly due to the fact that the infection of endophyte might stimulate the production of internal hormones.

Our experiments showed a higher content of soluble sugars in A. *inebrians* seeds from the Xiahe site. Studies showed that germination rate increases with the increase in soluble sugar content, suggesting that soluble sugars play a positive role in supporting seed germination [13,48,49]. Wen et al. [50] reported that carbohydrate is the storage substance of plants, responsible for maintaining tissues during chilling, while IAA plays a positive role in maintaining GA/ABA ratios.

## 5. Conclusions

In summary, the present study was designed to determine the effects of various seed dormancy-breaking treatments, seed origins and *Epichloë* endophyte infection in *Achnatherum inebrians* on seed germination. These experiments confirmed that the infection of endophyte stimulates the germination rate, content of soluble sugar, IAA, and GA of seeds from different geographic origins. Water soaking significantly increased seed germination rates. This is the first study to examine the associations among plant–geographic origins, dormancy break methods and endophytes. The analysis extended our knowledge of how endophytes and internal substances interact in seeds of *A*. *inebrians*. The relationship between *Epichloë* endophyte and endogenous hormone levels in seeds had positive effects on germination. We propose that the effects of endophyte on seed germination and seedling vigor should be considered in future turfgrass breeding efforts.

## Figures and Tables

**Table 1:** Origins of *Achnatherum inebrians* plant lineages and their attributes.

Location	Longitude and Latitude	Elevation (m)	Mean Temperature (°C)	Mean Precipitation (mm)
Gansu-Yuzhong (YZ)	35°56′26.7″ N	1850	6.6	320
	104°02′48.9″ E			
Gansu-Yingtai (YT)	36°52′42.7″ N	2279	5.3	331
	103°47′19.8″ E			
Gansu-Xiahe (XH)	35°32′16.4″ N	3500	2.6	516
	102°25′36.7″ E			
Ningxia-Haiyuan (HY)	36°36′9″ N	1732	7	286
	105°39′46″ E			
Alxa League (AL)	38°39′15″ N	1830	8	113
	105°44′21″ E			
Alxa Desert (ALD)	38°56′16″ N	1660	7.7	39
	105°47′39″ E			

**Table 2:** Experimental design of germination tests conducted in laboratory.

Break Dormancy Method	Immersion Time	Temperature
Needle pin (M1)	/	22 ± 2 °C
Water soaking (M2)	30 min	25/20 °C
98% H2SO4 soaking (M3)	20 min	22 ± 2 °C
Exogenous GA soaking(M4)	30 min	22 ± 2 °C
Control (CK)	/	22 ± 2 °C

**Table 3:** Effects of origin, endophyte, dormancy-breaking method, and their interaction on germination and content of soluble sugar, gibberellin, auxin, and abscisic acid in seeds of *Achnatherum inebrians*. Df = degree of freedom, F = f value is ratio of two means to effect/error term, *p* = significance.

Source of Variation	df	Germination Rate		Soluble Sugar		Gibberellin		Auxin		Abscisic Acid	
		F	*p*	F	*p*	F	*p*	F	*p*	F	*p*
Origin	5	53.01	<0.001	992.18	<0.001	455.01	<0.001	59.37	<0.001	19.18	<0.001
Method	4	438.88	<0.001	193.03	<0.001	1510.96	<0.001	35,819.20	<0.001	3909.35	<0.001
Endophyte	1	563.25	<0.001	62.07	<0.001	2.63	<0.05	1.55	<0.05	10.42	<0.001
Origin*Method	20	4.38	<0.001	3.13	<0.001	71.71	<0.001	1.67	0.22	2.51	<0.001
Origin*Endophyte	5	2.09	0.07	16.27	<0.001	0.99	0.42	1.11	0.36	3.95	<0.001
Method*Endophyte	4	10.68	<0.001	1.40	0.24	4.72	<0.001	0.63	0.65	2.06	0.09
Origin*Method*Endophyte	20	2.24	<0.001	0.77	0.74	1.70	<0.05	1.68	0.05	1.52	0.09

## Data Availability

Data available in a publicly accessible repository.

## Supplementary Materials

The following are available online at https://www.mdpi.com/article/10.3390/microorganisms9112183/s1, Figure S1: Germination rate of infected (E+) and non-infected (E−) Achnatherum inebrians from different treatments on an altitude (origin) gradient.
