# Supplementary material for: Interactive Effects of Epichloë Endophyte, Dormancy-Breaking Treatments and Geographic Origin on Seed Germination of Achnatherum inebrians
Source: Microorganisms. 2021 Oct 20;9(11):2183. doi: 10.3390/microorganisms9112183 (PMC8625081; doi:10.3390/microorganisms9112183)
Supplement: Supplementary file 1 [file microorganisms-09-02183-s001.zip › microorganisms-1392914-supplementary.pdf]

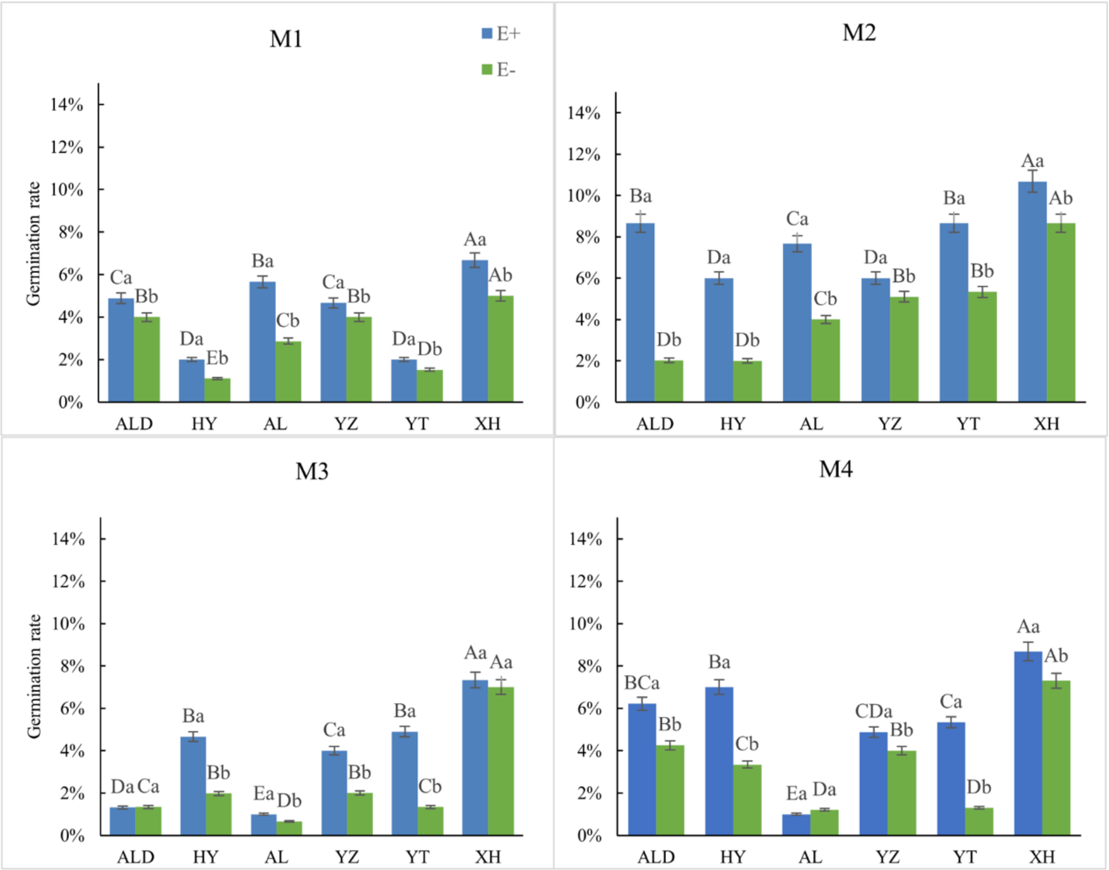

Figure S1. Germination rate of infected (E+) and non-infected (E−) *Achnatherum inebrians* from different treatments on an altitude (origin) gradient. The different capital letters indicate the mean significant difference ($p < 0.05$) in endophyte status (E+ or E−) in seed germination under different origin. The different lowercase letters indicate the mean significant difference ($p < 0.05$) in the pair-wise comparisons of endophyte status (E+ and E−) in seed germination in the same origin.
